# Sleep Disturbance in Bipolar Disorder. Treatment Implications

**DOI:** 10.1192/j.eurpsy.2024.902

**Published:** 2024-08-27

**Authors:** T. Jupe, A. Roumpou, K. Provi

**Affiliations:** Psychiatric Hospital of Attica, Athens, Greece

## Abstract

**Introduction:**

Relationship between sleep and bipolar disorder involves the following aspects: decreased need for sleep is a fundamental marker of the manic state, sleep deprivation is one cause of mania and may in fact be a fundamental etiological agent in mania, total sleep time is a predictor of future manic episodes, and total sleep time may be a marker of response as well as a target of treatment in mania.

**Objectives:**

This e-poster aimed to summarize evidence regarding the sleep disturbance in Bipolar Disorder.

**Methods:**

Bibliopgraphical review was performed using PubMed platform. All relevant articles were found using the keywords: sleep disturbance, bipolar disorder, mania.

**Results:**

Sleep disturbances are frequent in BD patients in different phases of illness, including the euthymic state and remission. These sleep aberrations are represented not only by insomnia but also by sleep–wake rhythm disorders, especially delayed sleep–wake phase disorders. During the manic state, most patients experience a reduced need for sleep and longer sleep onset latency. Likewise, in the depressive state, insomnia and hypersomnia are commonly observed. Meta-analyses of trials conducted on remitted BD patients demonstrated prolonged total sleep time, increased awakenings after sleep onset, greater variability of sleep–wake variables, and reduced sleep efficiency.

**Conclusions:**

Overall, all kinds of sleep disorders and parasomnias are very common especially in youth patients with BD. Thus, compared to the general population, youth with BD exhibit lower sleep efficiency, longer slow wave sleep, and reduced REM sleep, features that could affect the genesis and prognosis of the disorder. Sleep disturbances may also be used as predictors of the onset of BD in a subset of high-risk young subjects.

**Disclosure of Interest:**

None Declared

